# The influence of “small private online course + flipped classroom” teaching on physical education students’ learning motivation from the perspective of self-determination theory

**DOI:** 10.3389/fpsyg.2022.938426

**Published:** 2022-08-23

**Authors:** Ti Hu, Meng-long Zhang, Hong Liu, Jun-cheng Liu, Si-jia Pan, Jiang-hao Guo, Zong-en Tian, Lei Cui

**Affiliations:** ^1^College of Physical Education and Sports, Beijing Normal University, Zhuhai, China; ^2^Zhengzhou No. 17 Senior Middle School, Zhengzhou, China; ^3^Shenzhen Senior High School Group, Shenzhen, China

**Keywords:** “SPOC + flipped classroom” teaching, self-determination theory, learning motivation, influencing factors, internalization, physical education student

## Abstract

**Objective:**

The study aimed to enhance the learning motivation of college physical education students and improve their learning outcomes. Based on the perspective of the self-determination theory, this study explores the influence of “Small Private Online Course (SPOC) + flipped classroom” teaching on the learning motivation of students majoring in physical education and profoundly analyzes the influencing factors and promotion paths of learning motivation using this model.

**Materials and methods:**

A total of four classes (64 students) of physical education majors in a university were selected and randomly divided into an experimental group (34 students) and a control group (30 students). The experimental group received “SPOC + flipped classroom” teaching, the control group received traditional teaching. Before and after the 16-week intervention, learning motivation, teacher support perception, basic psychological need satisfaction, and academic emotions of the 64 students were measured, and the data were analyzed by repeated-measures analysis of variance and partial least square regression.

**Results:**

(1) The instructional intervention reduced non-regulation, external regulation, and introjected regulation, while increased identified regulation, intrinsic regulation, and self-determination levels in the students. The levels of non-regulation, external regulation, identified regulation, and self-determination were also significantly different from those of the control group. (2) After the intervention, the scores of support for autonomy, support for competence, support for relatedness, and need for relatedness in the experimental group were significantly higher than those in the control group. (3) Support for autonomy, support for competence, support for relatedness, need for competence and need for relatedness positively predicted the self-determination level, and intrinsic regulation and identified regulation negatively predicted non-regulation, external regulation, and introjected regulation.

**Conclusion:**

“SPOC + flipped classroom” teaching has a positive impact on students’ learning motivation of basketball skills and promotes students’ motivation autonomy. The improvement of support for autonomy, support for competence, support for relatedness, need for competence, and need for relatedness may be related to the improvement of learning motivation of college students majoring in Physical Education (PE). “SPOC + flipped classroom” teaching enables students to obtain more demand satisfaction by giving them more demand support, while demand support and demand satisfaction can promote the internalization of learning motivation so that students can maintain high autonomy motivation.

## Introduction

Since the beginning of the 21st century, with the rapid development of information technology, an increasing number of countries have embarked on educational informatization (Yang et al., 2014). Network teaching arises at this historic moment, and flipped classrooms have developed rapidly and attracted global attention. In 2012, MOOC prompted intense discussions around the world, and a large number of high-quality open online courses emerged, further promoting the rapid development of online education and blended teaching. Xu et al. (2014) proposed small private online course (SPOC), considered “post-MOOC”, to give full play to the role of online courses more efficiently. Small Private Online Course (SPOC), supported by advanced education and information technology, changes the time and space limitations of the traditional teaching model, provides rich learning materials for students to communicate and collaborate anytime and anywhere, realizes an open educational environment, and brings together the advantages of online courses and face-to-face teaching, which also provides a strong guarantee of flipped classroom knowledge acquisition (De La Croix and Egerstedt, 2014). The combination of flipped classrooms and SPOC will bring new vitality to course teaching (Gu et al., 2017). In terms of “SPOC + flipped classroom” teaching design, the primary forms in other disciplines are self-study teaching videos before class to absorb knowledge, diverse teaching activities in class to help students complete the internalization of knowledge, and consolidation and feedback through an online platform after class (Li and Li, 2015; Peng and Long, 2020). The design of physical education is essentially the same as that of other subjects, but due to the physical activity-based nature of the physical education classroom, the specific implementation forms are different, with classroom activities primarily based on group cooperation, demonstration, and intergroup competition (Hinojo-Lucena et al., 2018; Peng and Long, 2020). In addition, due to the more difficult mastery of motor skills and the longer learning cycle, consolidation exercises and feedback on questions in the post-class phase have been emphasized (Wang et al., 2019). In the teaching practice “SPOC + flipped classroom” model, it has been found that it helps students master technical actions and relevant theoretical knowledge and significantly promotes learning attitudes, learning efficiency, enthusiasm, motivation, and self-efficacy (Wang et al., 2019; Liu, 2020), but there are relatively few studies on students’ learning motivation, and only simple comparisons are made at a superficial level (Kurt, 2017; Wang, 2021), and there is a lack of detailed discussion on the factors and mechanisms influencing learning motivation.

Motivation is the internal psychological tendency that causes and maintains individual behavior to meet its needs and is the power source of individual behavior. Learning motivation is the psychological tendency and motivation source of individuals to produce and continue learning behavior, cause the individuals’ learning behavior and maintain and continue it, and is the internal motivation of the individuals to participate in learning (Pi et al., 2009). Relevant studies have shown that insufficient motivation to learn can hinder the improvement of the teaching effectiveness of physical education courses (Su, 2007), while greater motivation promotes the improvement of the learning effect (Yang, 2021). At present, college students majoring in physical education are in a state of high cognition and low desire for professional learning (Zhou, 2010), with weak learning motivation and engagement (Luo et al., 2017; Mao, 2020); as the grades increase, learning burnout is becoming an increasingly serious problem, which reduces the learning effect (Zhang, 2010; Liu et al., 2016). Therefore, it is crucial to explore new teaching modes and enhance the learning motivation of physical education students in order to improve the teaching effect and the quality of talent training.

The self-determination theory (SDT), developed by Deci and Ryan (1985), systematically explains the continuous structure, regulation model, influencing factors, and mechanism of motivation and puts forward the main viewpoints of *basic psychological needs*, organic integration, causal orientation, and *cognitive evaluation*. In exploring learning motivation, the self-determination theory states that individual motivation is on the continuum of *no motivation*, *external motivation* (multiple adjustment methods), and *internal motivation* (Table 1) and that the higher the degree of self-determination of motivation, the greater the autonomy of motivation (Deci and Ryan, 2000). In terms of exploring the factors affecting learning motivation, the SDT holds that the social environment influences the internalization of motivation by influencing the satisfaction of three basic psychological needs (*need for competence*, *need for autonomy*, and *need for relatedness*) of individuals. Relevant research shows that the satisfaction of basic psychological needs significantly predicts the internalization of *internal motivation* and *external motivation* (Joe et al., 2017), and the task of meeting individual basic psychological needs can significantly affect *internal motivation*. Second, the SDT holds that the outside world affects motivation through individuals’ cognitive evaluation of external events, that is, the external support (*support for autonomy*, *support for relatedness*, and *support for competence*) felt by students impacts motivation. Reeve (2002) uses the SDT to explain the process of learning motivation that when teachers provide *support for autonomy*, students can benefit from it, and it improves students’ learning motivation. Another study shows that *support for autonomy* can promote the development of *identified regulation* and *intrinsic regulation* (Deci and Ryan, 2000). In addition, emotions (*positive activity orientation*, *positive outcome orientation*, *negative activity orientation*, and *negative outcome orientation*) are the source of motivation for self-determination, which plays a vital role in learning motivation (Qiao and Li, 1995; Li et al., 2004). Studies have shown a significant positive correlation between positive emotions and learning motivation variables, and a significant inverse correlation between negative emotions and learning motivation variables (Jin, 2021). Therefore, this study measures the degree of self-psychological needs, external support, and academic emotions that students feel and explores their connection to learning motivation.

**TABLE 1:** Explanation of different forms of motivation regulation.

	Adjust the way	Meaning
Amotivation	Non-Regulation	Individuals cannot be motivated when they do not recognize the connection between their behavior and the desired outcome;
Extrinsic motivation	External Regulation	Reflects that the individual is under the control of external events (such as rewards and punishments) to take a certain behavior, has the lowest autonomy.
	Introjected Regulation	Individuals adjust their own behaviors through the perceived values and reflect external motivation to begin to internalize and have a certain degree of autonomy.
	Identified Regulation	Individuals evaluate events or behaviors, recognize their value to themselves, and then decide whether to take action; However, there is no spontaneous pleasure or satisfaction.
Intrinsic motivation	Intrinsic Regulation	Internal motivation is derived from the tendency to grow and develop mentally and is caused by the satisfaction generated by the behavior itself, without the involvement of external conditions.
	Self-determination level	The comprehensive expression of motivation level represents students’ self-determination level, and the higher the score, the more independent it is.

Based on this, from the perspective of the self-determination theory, this study explores the influence of “SPOC + flipped classroom” teaching on the learning motivation of students majoring in physical education and profoundly analyzes the influencing factors and promotion paths of learning motivation under this model. The research is mainly carried out from the following three aspects:

1. The impact of “SPOC + flipped classroom” teaching on the learning motivation of physical education students.

2. Factors influencing the learning motivation of physical education students under the teaching of “SPOC + flipped classroom.”

3. The “SPOC + flipped classroom” teaching affects the promotion paths of the learning motivation of physical education students.

## Materials and methods

### Subjects and study design

In the autumn semester of 2020, four classes (64 students in total) of physical education majors in a university in Beijing were selected for the basketball teaching experiment, and the four classes were randomly divided into an experimental group (34 students) and a control group (30 students). A mixed experimental design of 2 (experimental group and control group) × 2 (pretest and posttest) was used. The experimental group adopted “SPOC + flipped classroom” teaching, while the control group adopted the traditional teaching approach; the experiment lasted for one semester (16 weeks). Before and after the experiment, students’ learning motivation and teachers’ perceptions of support, basic psychological need satisfaction, and academic emotions were measured in both groups.

### “SPOC + flipped classroom” teaching design

This study is based on the following aspects: three dimensions of basketball course teaching objectives, learner characteristics, and model characteristics; two platforms of SPOC and classroom; two perspectives of teacher and student; three stages, namely before class (knowledge transfer), in class (knowledge internalization), and after class (knowledge consolidation), to carry out the “SPOC + flipped classroom” basketball teaching design. In the before class stage, teachers publish the learning resources of this class on the SPOC platform. Students learn relevant theoretical knowledge about basketball skills and tactics by themselves (action methods, tactics, rules, etc.) and complete online tests. Then, through cognitive imitation of skills and tactics and peer training, the visual representation was initially established. Finally, a real-time communication channel is established through the online platform to feed back problems that are difficult to solve for teachers. In the class stage, teachers target guidance in doubts and difficulties in students before class learning, solving students’ questioning and corrective technical actions, thereby shortening the time of explaining the primary content. Through the organization, collaboration, dialog, competition, and other organizations, students’ enthusiasm for learning is fully mobilized so that students will continue to internalize and consolidate the content of the learning in the process of high-density “learning,” “practice,” and “competition.” At the after class stage, the teachers arranged the after-school learning tasks through the SPOC platform, answered doubts for students, and completed the tracking and evaluation of students’ learning effects in the process. Students have practiced autonomous exercises, group exercises, and practical applications and jointly completed after-class tasks. At the same time, after class feedback also provides reference and guidance for before class teaching to achieve an adequate grasp and comprehensive application of knowledge and skills.

### Data collection

#### Measurement of learning motivation

The Perceived Locus of Causality Scale was used to estimate students’ learning motivation level. The scale, developed by Goudas et al. (1994), is suitable for measuring the motivation and behavioral regulation of middle school students in physical education and sports, and it was later revised and introduced in China by Pak-Kwong et al. (2014) and has good reliability and validity with five dimensions: *intrinsic regulation*, *identified regulation*, *introjected regulation*, *external regulation*, and *non-regulation*. In this study, Cronbach’s α coefficients were 0.81, 0.73, 0.64, 0.71, and 0.77, respectively, and the total table coefficients were 0.82. The self-determination index (SDI) is calculated in the form of weighting as follows: 2 × *intrinsic regulation* + *identified regulation* - *introjected regulation* -2 × *external regulation*; the higher the score, the more autonomous it is, and the more inclined the learning behavior is toward a *self-determination level.* This index does not include the dimension of *amotivation* because the SDI represents the *self-determination level* of individual motivation, while *amotivation* represents that individuals have no motivation to stimulate, and its effectiveness has been verified in many studies (Grolnick and Ryan, 1987; Vallerand et al., 1997; Standage et al., 2006).

#### Measurement of physical education needs support

The Perceived Need Support Scale in physical education was used to evaluate students’ perceived need for support. This questionnaire, which was compiled by Yin et al. (2018), is suitable for measuring the perception of demand support in PE teaching. It contains three dimensions, *support for autonomy*, *support for competence*, and *support for relatedness*, and has high reliability and validity. Cronbach’s α coefficients in this study were 0.97, 0.93, and 0.96, respectively, and the total table coefficient was 0.99.

#### Measurement of academic emotion

The General Academic Emotion Questionnaire for College Students (GAEQ) was used to evaluate students’ academic emotion. This questionnaire, compiled by Xu and Gong (2011), is suitable for evaluating college students’ academic emotions, and it contains four subscales of *positive activity orientation*, *positive outcome orientation*, *negative activity orientation*, and *negative outcome orientation* and has good reliability and validity. Cronbach’s α coefficients of each subscale in this study were 0.92, 0.94, 0.95, and 0.85, respectively, and the coefficient of the total scale was 0.94.

#### Measurement of basic psychological needs

Basic psychological needs were measured by the questionnaire used by (Zhu et al., 2011) in this questionnaire, *need for autonomy* was measured by the relevant scale in Hollembeak and Amorose (2005), *need for competence* was measured using the relevant scale in the *intrinsic motivation inventory* (IMI) of McAuley et al. (1989); and *need for relatedness* was measured using the relevant scale in *the Need for Relatedness Scale* (NRS) developed by Richer and Vallerand (1998). The three subscales have been widely used and certified in the field of sports. Through confirmatory factor analysis, Zhu Xiaona showed that the reliability and validity of the three questionnaires were good. In this study, Cronbach’s α coefficients of the three subscales of competence, autonomy, and relationship were 0.86, 0.71, and 0.98, respectively, and the total coefficient was 0.96.

### Data analysis

In this study, SPSS 22.0 and SIMCA-P 11.5 software were used to conduct *analysis of variance* (ANOVA) and *partial least square regression* (PLSR) analyses to explore the influence of different models on students’ motivation, as well as their influencing factors and mechanisms. PLSR analysis with principal component analysis, canonical correlation analysis, and multiple linear regression of some of the common characteristics are able to analyze large numbers of variables in small sample sizes. Thus, considering the small number of samples and a large number of variables in this study, we performed the PLSR analysis. PLSR analysis mainly includes the following two steps: first, identify the number of principal components; second, after setting the number of principal components for specific analysis, (1) analyze the relationship expression, correlation coefficient, and accuracy between principal components and research items; (2) analyze the influence relationship between independent variables and dependent variables; and (3) projection importance analysis.

## Research results

### Influence of “SPOC + flipped classroom” on learning motivation

Table 2 shows the descriptive statistical results of the motivation level and *self-determination level* of each dimension of the experimental group and the control group before and after the teaching intervention. Compared with before the intervention, the overall level of the experimental group increased after the intervention, while that of the control group decreased. A one-way ANOVA results showed that there was no significant difference between the pretest group and the control *group (self-determination level* (*F*_(1_, _62)_ = 0.569, *p* = 0.453 > 0.05, *partial* η*^2^* = 0.009); *non-regulation* (*F*_(1_, _62)_ = 3.175, *p* = 0.080 > 0.05, *partial* η*^2^* = 0.049); *external regulation* (*F*_(1_, _62)_ = 1.183, *p* = 0.281 > 0.05, *partial* η*^2^* = 0.019); *introjected regulation* (*F*_(1_, _62)_ = 0.998, *p* = 0.322 > 0.05, *partial* η*^2^* = 0.016); *identified regulation* (*F*_(1_, _62)_ = 0.566, *p* = 0.455 > 0.05, *partial* η*^2^* = 0.009); and *intrinsic regulation (F*_(1_,_62)_ = 0.032, p > 0.05, *partial* η*^2^* = 0.001). Next, the influence of teaching intervention on students’ motivation is analyzed.

**TABLE 2:** Motivation levels of the experimental group and the control group before and after intervention.

Learning motivation	The experimental group				The control group			
	Before the test		After the test		Before the test		After the test	
	M	SD	M	SD	M	SD	M	SD
Self-determination level	8.5200	4.97092	9.9021	4.81957	7.5887	4.87984	6.4333	5.06685
Non-regulation	2.0588	1.24644	1.7844	1.23039	2.6893	1.58061	2.7773	1.71609
External regulation	2.8526	1.60206	2.5294	1.11049	3.2780	1.51396	3.3557	1.61886
Introjected regulation	3.8732	1.70758	3.6668	1.76412	4.2787	1.51399	4.1443	1.58696
Identified regulation	5.8426	1.24790	6.1176	0.94901	6.0670	1.12227	5.6450	1.16111
Intrinsic regulation	6.1279	1.20581	6.2553	0.90605	6.1777	0.98913	5.8223	1.23698

**FIGURE 1:**
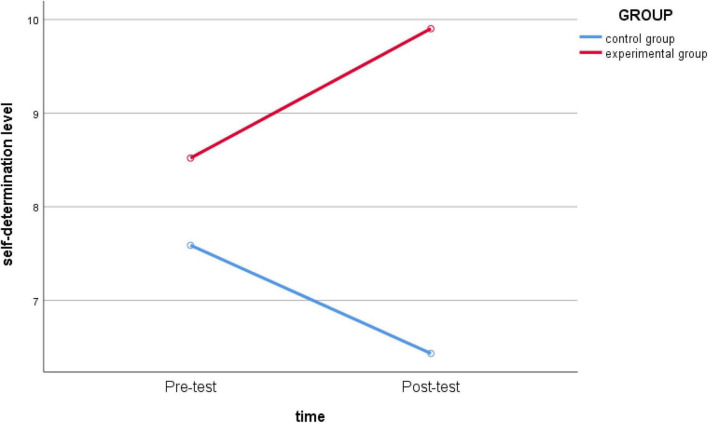
Schematic diagram of the changes in *self-determination level* between the experimental group and the control group before and after intervention.

In the dimension of self-determination level, a repeated-measures ANOVA was conducted for intra- and intrasubject effect tests (Table 3), indicating that the main effect of time was not significant (*F*_(1_,_62)_ = 0.018, *p* = 0.894 > 0.05, *partial* η*^2^* = 0.000), that is, there was no significant difference in the *self-determination level* of each group over time. The interaction effect of time * group was not significant (*F*_(1_,_62)_ = 2.243, *p* = 0.139 > 0.05, *partial* η*^2^* = 0.035), indicating that there was no significant difference in the change in the se*lf-determination level* between the experimental group and the control group before and after intervention. Further post-analysis of the group’s main effect showed that there was no significant difference between the experimental group and the control group, and the *self-determination level* of the experimental group was significantly higher than that of the control group (*F*_(1_,_62)_ = 5.982, *p* = 0.017 < 0.05, *partial* η*^2^* = 0.088) (Figure 1).

**TABLE 3:** Detection of intra- and intersubject effects.

Measure: MEASURE_1					
The source	Class III sum of squares	Degrees of freedom	The mean square	F	Significant
Time	0.410	1	0.410	0.018	0.894
Time * group	51.306	1	51.306	2.243	0.139
Group	154.279	1	154.279	5.982	0.017*

*p < 0.05.

**FIGURE 2:**
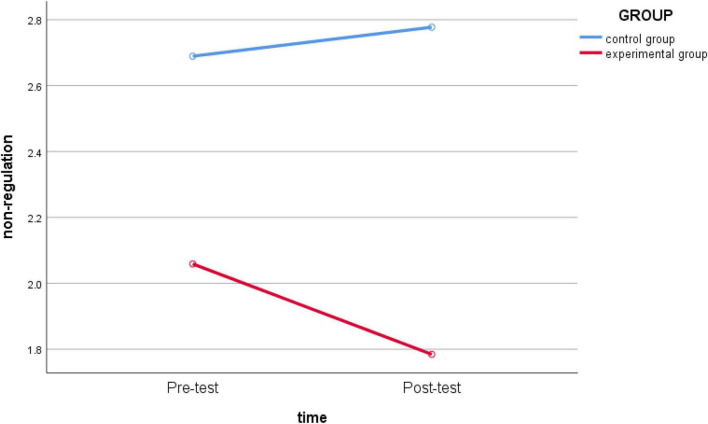
Schematic diagram of the changes in *non-regulation* between the experimental group and the control group before and after intervention.

**FIGURE 3:**
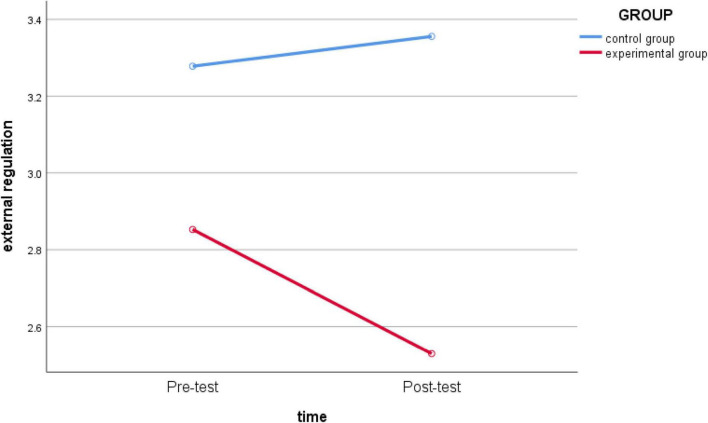
Schematic diagram of the changes in *external regulation* between the experimental group and the control group before and after intervention.

**FIGURE 4:**
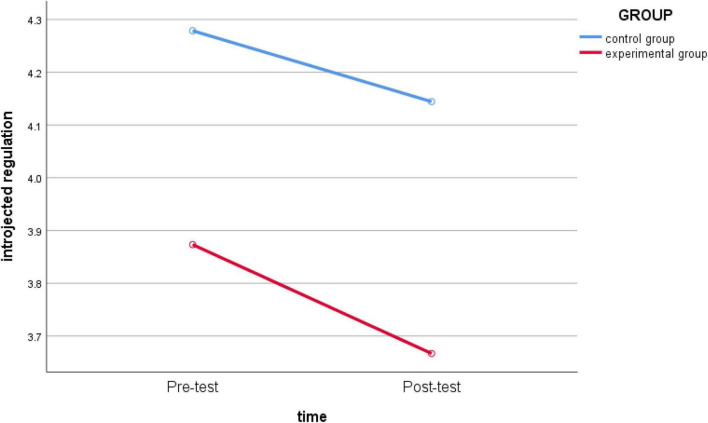
Schematic diagram of the changes in *introjected regulation* between the experimental group and the control group before and after intervention.

**FIGURE 5:**
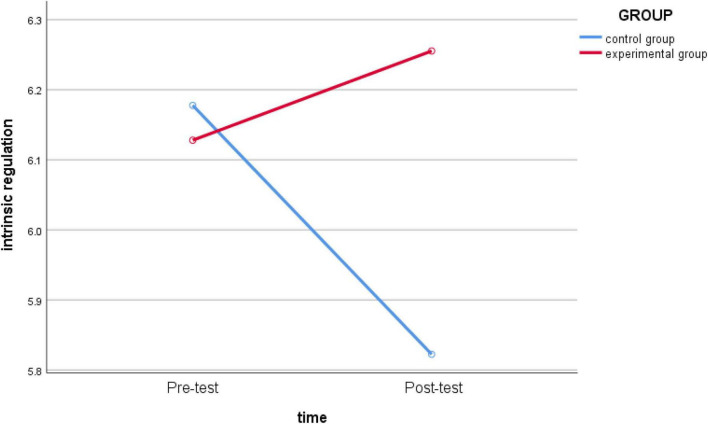
Schematic diagram of changes in *intrinsic regulation* between the experimental group and the control group before and after intervention.

**FIGURE 6:**
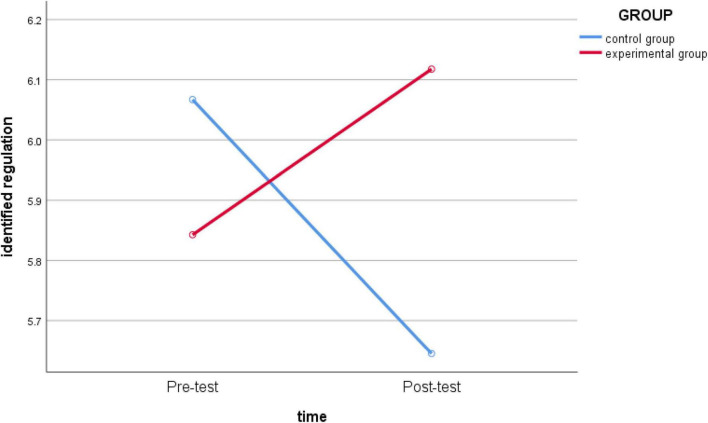
Schematic diagram of the changes in *identified regulation* between the experimental group and the control group before and after intervention.

Then, the repeated measures ANOVA on the five dimensions of non-regulation, external regulation, introjected regulation, identified regulation, and intrinsic regulation. The repeated-measures ANOVA results showed *non-regulation* (*F*_(1_,_62)_ = 10.533, *p* = 0.002 < 0.05, *partial* η*2* = 0.145) (Figure 2) and *external regulation* (*F*_(1_,_62)_ = 5.244, *p* = 0.025 < 0.05, *partial* η*2* = 0.078) (Figure 3); the main effect of the two-dimensional group was significant, indicating that teaching intervention had a significant impact. The group effects of *introjected regulation* (*F*_(1_,_62)_ = 1.584, *p* = 0.213 > 0.05, *partial* η*2* = 0.025) (Figure 4) and *intrinsic regulation* (*F*_(1_,_62)_ = 0.806, *p* = 0.373 > 0.05, *partial* η*2* = 0.013) (Figure 5) were not significant, indicating that there was no significant difference in the level of different groups. Among them, the group effect of *identified regulation* was not significant, but the interaction effect of time* group was significant (*F*_(1_,_62)_ = 4.137, *p* = 0.046 < 0.05, *partial* η*2* = 0.063) (Figure 6). Further analysis showed that there were significant differences in the degree of change in the *identity regulation* level before and after intervention for different groups, and the experimental group had a positive influence on the identity regulation level, while the control group experienced the opposite effect.

Next, we conducted a *post hoc* power analysis using software G*Power (version 3.1.9.2; Kiel University, Kiel, Germany) to confirm the sample sizes. We used a power analysis with an effect size f = 0.2592 or 0.4118 (*partial* η*2* = 0.063 or 0.145), α error of probability = 0.05, total sample size = 64, number of groups = 2, number of measurements = 2, correlation = 0.5, and power (1-β) = 0.98 or 0.99.

As a result, the instructional intervention reduced *non-regulation*, *external regulation*, and *introjected regulation*, while increased *identified regulation*, *intrinsic regulation*, and *self-determination levels* in the students. The levels of *non-regulation*, *external regulation*, *identified regulation*, and *self-determination* were also significantly different from those of the control group.

### Partial least square regression analysis of the influencing factors of “SPOC + flipped classroom” teaching to improve learning motivation

#### Determine the number of principal components

In this study, cross-validity analysis was used to determine the number of principal components, supplemented by importance in projection Variable Importance in Projection (VIP) value analysis. Crossover validity can be used to analyze the optimal number of components. If Qh2 ≤ 0.0975, it is meaningless to increase the number of principal components further, that is, the number of components corresponding to this point (or the upper point) is the optimal number of principal components. When *h* = 1, Qh2 = 1.000 > 0.0975; therefore, it is suitable to extract one principal component (Table 4).

**TABLE 4:** Results of cross-validation analysis.

Composition h	SS	PRESS	Qh^2^
1	494.728	562.397	1.000
2	474.789	595.170	−0.203
3	466.268	596.233	−0.256
4	455.139	702.616	−0.507
5	449.329	719.839	−0.582
6	427.953	753.313	−0.677
7	423.360	753.947	−0.762
8	420.341	984.184	−1.325
9	383.388	958.450	−1.280
10	381.861	956.701	−1.495

The VIP value was further analyzed. By comparing the VIP values of the projected importance of principal components with different numbers, it was found that there was no significant difference between the VIP values of each variable when there were one principal component and multiple principal components. Combined with the results of the cross-validity analysis, the number of principal components was finally determined to be 1.

#### Partial least square regression analysis

In PLSR analysis, multiple independent variables and dependent variables will be concentrated to represent the principal component U and principal component V, which are then used as bridges for research. Through analysis, the relationship expression between principal components U and V and the variables is obtained as follows: (1) U1 = 0.427*support for autonomy + 0.407* *support for relatedness* + 0.429* *support for competence* + 0.347* *need for competence* + 0.186* *need for autonomy* + 0.404* *need for relatedness* + 0.267* *positive activity orientation* + 0.261* *positive outcome orientation* −0.038* *negative activity orientation* + 0.096* *negative outcome orientation.* (2) V1 = −0.571* *non-regulation* −0.544* *external regulation* −0.167* *introjected regulation* + 0.663*identified regulation + 0.755* *intrinsic regulation* + 0.726* *self-determination level.*

The factor loading value between principal components and research items is used to analyze the correlation between the principal components and analysis items, the value is between -1 and 1, and the larger the absolute value, the stronger the correlation. Table 5 shows that there is a positive correlation between the principal component U1 and the respective variables. The principal component V1 was negatively correlated with *non-regulation*, *external regulation*, and *introjected regulation* and positively correlated with *identified regulation*, *intrinsic regulation*, and *self-determination levels.* Further analysis of principal components U1 and V1 and the accuracy of the research item shows that the extracting ratio of principal component U to the information of the 10 independent variables is 0.546 (i.e., the variance explanation rate is 54.6%), which is acceptable. Among them, the information extraction proportion of *support for autonomy*, *support for relatedness*, *support for competence*, *need for competence*, *need for competence*, and *need for autonomy* was very high (0.906, 0.820, 0.861, 0.719, and 0.837, respectively), while the information extraction proportion of *need for autonomy*, *positive activity orientation*, and *positive outcome orientation* was low (0.333, 0.406, and 0.432, respectively); the proportion of information extraction *for negative activity orientation* and *negative outcome orientation* was very low (0.004 and 0.141, respectively). The extraction ratio of principal component V1 to the six dependent variables was 0.597 (i.e., the variance explanation rate was 59.7%), which was acceptable. Among them, the proportion of information extraction of *non-regulation*, *external regulation*, *identified regulation*, *intrinsic regulation*, and *self-determination* level was very high (0.558, 0.648, 0.616, 0.745, 0.924), while the proportion of information extraction of *introjected regulation* was very low (0.092).

**TABLE 5:** Correlation analysis between principal components and research items (loading value).

	Principal component U1
Support for autonomy	0.410
Support for relatedness	0.390
Support for competence	0.400
Need for competence	0.366
Need for autonomy	0.249
Need for relatedness	0.395
positive activity orientation	0.275
Positive outcome orientation	0.284
Negative activity orientation	0.029
Negative outcome orientation	0.162
	**Principal component V1**
Non-regulation	−0.261
External regulation	−0.249
Introjected Regulation	−0.076
Identified Regulation	0.303
Intrinsic regulation	0.345
Self-determination level	0.332

Table 6 shows the standardized regression expression between the dependent variable Y and independent variable X, without motivation: (1) *non-regulation* = -0.111*support for autonomy-0.106* *support for relatedness* -0.112*support for competence-0.091*need for competence-0.049*need for autonomy −0.106*need for relatedness-0.070*positive activity orientation-0.068*positive outcome orientation + 0.010*negative activity orientation −0.025*negative outcome orientation. The other five dimensions are the same. The result shows that the *self-determination level*, *intrinsic regulation*, and *identified regulation*, in addition to *negative activity orientation*, have a negative impact. *Support for autonomy*, *support for relatedness*, *support for competence*, *need for competence*, *need for autonomy*, *need for relatedness*, *positive activity orientation*, *positive outcome orientation*, and *negative outcome orientation* have a positive influence on the results. Among them, the influence of *support for autonomy*, *support for relatedness*, *support for competence*, *need for competence*, and *need for relatedness* is larger, followed by the influence of *positive activity orientation* and *positive outcome orientation*, and the impact of *need for autonomy*, *negative activity orientation*, and *negative outcome orientation* is smaller. *Non-regulation* and *external regulation*, in addition to *negative outcome orientation*, have a positive impact. *Support for autonomy*, *support for relatedness*, *support for competence*, *need for competence*, *need for autonomy*, *need for relatedness*, *positive activity orientation*, *positive outcome orientation*, and *negative outcome orientation* all have a negative impact on them. Among them, *support for autonomy*, *support for relatedness*, *support for competence*, and *need for relatedness* have a greater impact, followed by the influence of *need for competence*, *positive activity orientation*, and *positive outcome orientation*, and the impact of *need for autonomy*, *negative activity orientation*, and *negative outcome orientation* is small. I*ntrojected regulation*, in addition to *negative outcome orientation*, has a positive impact. *Support for autonomy*, *support for relatedness*, *support for competence*, *need for competence*, *need for autonomy*, *need for relatedness*, *positive activity orientation*, *positive outcome orientation*, and *negative outcome orientation* all have a negative impact, but the impact of all independent variables is less.

**TABLE 6:** Regression coefficients between dependent variable Y and independent variable X.

	Not standardized						Standardized					
	Non- regulation	External regulation	Introjected regulation	Identified regulation	Intrinsic regulation	Self-determination level	Non-regulation	External regulation	Introjected regulation	Identified regulation	Intrinsic regulation	Self-determination level
Constant	7.854	7.723	6.406	1.363	0.832	−18.825	0.000	0.000	0.000	0.000	0.000	0.000
Support for autonomy	−0.169	−0.145	−0.071	0.151	0.164	0.841	−0.111	−0.106	−0.033	0.129	0.147	0.142
Support for relatedness	−0.165	−0.142	−0.069	0.148	0.160	0.821	−0.106	−0.101	−0.031	0.124	0.141	0.135
Support for competence	−0.150	−0.129	−0.063	0.135	0.146	0.749	−0.112	−0.107	−0.033	0.130	0.148	0.142
Need for competence	−0.094	−0.081	−0.040	0.085	0.092	0.470	−0.091	−0.086	−0.027	0.105	0.120	0.115
Need for autonomy	−0.047	−0.040	−0.020	0.042	0.046	0.234	−0.049	−0.046	−0.014	0.056	0.064	0.062
Need for relatedness	−0.140	−0.121	−0.059	0.126	0.136	0.698	−0.106	−0.101	−0.031	0.123	0.140	0.134
Positive activity orientation	−0.008	−0.006	−0.003	0.007	0.007	0.038	−0.070	−0.066	−0.020	0.081	0.092	0.089
Positive outcome orientation	−0.015	−0.013	−0.006	0.014	0.015	0.077	−0.068	−0.065	−0.020	0.079	0.090	0.087
Negative activity orientation	0.001	0.001	0.000	−0.001	−0.001	−0.004	0.010	0.010	0.003	−0.012	−0.013	−0.013
Negative outcome orientation	−0.003	−0.002	−0.001	0.002	0.003	0.013	−0.025	−0.024	−0.007	0.029	0.033	0.032

The projected importance index VIP is used to explain the overall importance of the independent variable X to the dependent variable Y (Table 7). *Support for autonomy*, *support for relatedness*, *support for competence*, *need for competence*, and *need for relatedness* have greater explanatory power for students’ learning motivation (VIP value > 1.0), followed by *positive activity orientation* and *positive outcome orientation* (0.844 and 0.825, respectively). *Need for autonomy*, *negative activity orientation*, and *negative outcome orientation* had low explanatory power for students’ learning motivation (0.589, 0.121, and 0.303, respectively). Some variables contribute less to the regression model and need to be adjusted. To further determine the variables that need to be adjusted, combined with the regression coefficient test of the original data, the influence of individual independent variables on the dependent variables is not significant. Finally, the regression model was adjusted based on the standardized regression coefficient, VIP value, and regression coefficient test results.

**TABLE 7:** Summary of important indicators for projection (VIP).

Variable	VIP value
Support for autonomy	1.349
Support for relatedness	1.289
Support for competence	1.355
Need for competence	1.097
Need for autonomy	0.589
Need for relatedness	1.279
Positive activity orientation	0.844
Positive outcome orientation	0.825
Negative activity orientation	0.121
Negative outcome orientation	0.303

#### Partial least square regression model after adjustment

Through multiple variable adjustments, this study found that the model was ideal after removing the items *positive activity orientation*, *positive outcome orientation*, *negative activity orientation*, *negative outcome orientation*, and n*eed for autonomy*. The following is the adjusted analytical regression result.

The mathematical relationship between the adjusted principal components and the research items was as follows: (1) principal component U1 = 0.472* *support for autonomy* + 0.452* *support for relatedness* + 0.475* *support for competence* + 0.384* *need for competence* + 0.446* *need for relatedness*; (2) principal component V1 = −0.547* *non-regulation* −0.515* *external regulation* −0.167* *introjected regulation* + 0.542* *identified regulation* + 0.656* *intrinsic regulation* + 0.652* *self-determination level*. There is a positive correlation between the adjusted principal component U1 and the respective variables. Principal component V1 was negatively correlated with *non-regulation*, *external regulation*, and *introjected regulation* and positively correlated with *identified regulation*, *intrinsic regulation*, and *self-determination level*. After adjustment, the loading value is higher, and the correlation is stronger. The information extraction ratio of the adjusted principal component U1 to the respective variables reached 0.869 (i.e., the variance explanation rate was 86.9%), which was significantly higher than the previous rate of accuracy, and the extraction effect was ideal. The extraction ratio of principal component V1 to the information of each variable reached 0.599 (i.e., the variance explanation rate was 59.9%), which was relatively ideal.

Table 8 shows the regression expression between the dependent variable Y and independent variable X, including the relationship expression between each dependent variable Y and all independent variables, as explained later without motivation. The other five dimensions are the same: (1) *non-regulation* = −0.148*support for autonomy−0.142*support for relatedness −0.149* *support for competence* −0.121*need for competence−0.140*need for relatedness. The normalized values of the adjusted regression coefficients showed that *support for autonomy*, *support for relatedness*, *support for competence*, *need for competence*, and *need for relatedness* had positive effects on the *self-determination level*, *intrinsic regulation*, and *identified regulation*. In terms of *non-regulation* and *external regulation*, *support for autonomy*, *support for relatedness*, *support for competence*, *need for competence*, and *need for relatedness* all had negative effects. In terms of introjected *regulation*, *support for autonomy*, *support for relatedness*, *support for competence*, *need for competence*, and *need for relatedness* all had negative effects. However, all the independent variables have little influence. After adjustment, the VIP values of each variable are all greater than 0.8, which indicates a great influence on the dependent variable set, namely, learning motivation. Among them, the VIP value of *support for autonomy*, *support for relatedness*, and *support for competence* is greater than 1, which has a more important effect on learning motivation, while the effect of *need for competence* and *need for* relatedness on students’ motivation is relatively small.

**TABLE 8:** Adjusted regression coefficients between the dependent variable Y and independent variable X.

	Not standardized						Standardized					
	Non- regulation	External regulation	introjected regulation	identified regulation	Intrinsic regulation	Self-determination level	Non-regulation	External regulation	introjected regulation	identified regulation	Intrinsic regulation	Self-determination level
Constant	7.897	7.719	6.321	1.452	0.862	−18.584	0.000	0.000	0.000	0.000	0.000	0.000
Support for autonomy	−0.225	−0.191	−0.098	0.172	0.199	1.049	−0.148	−0.140	−0.045	0.147	0.178	0.177
Support for relatedness	−0.220	−0.187	−0.096	0.168	0.194	1.026	−0.142	−0.134	−0.043	0.141	0.170	0.169
Support for competence	−0.201	−0.170	−0.088	0.153	0.177	0.936	−0.149	−0.141	−0.045	0.148	0.179	0.178
Need for competence	−0.126	−0.107	−0.055	0.096	0.111	0.587	−0.121	−0.114	−0.037	0.120	0.145	0.144
Need for relatedness	−0.186	−0.158	−0.081	0.142	0.164	0.868	−0.140	−0.132	−0.043	0.139	0.168	0.167

To further test the fitting degree of the model, it can be seen from Table 9 that *R*^2^ = 0.438, that is, the set of independent variables can explain 42.8% of the reasons for the unmotivated changes. The other five dimensions are the same. The aforementioned results indicate that *non-regulation*, *external regulation*, *identified regulation*, *intrinsic regulation*, and *self-determination level* are good; *introjected regulation* is poor. On the one hand, this is related to the weak correlation between introjected regulation and other variables; on the other hand, it is also related to the information extraction ratio of principal component to introjected regulation is low.

**TABLE 9:** Summary of R-square model.

The regression model	R^2^
Non-regulation	0.428
External regulation	0.380
Introjected Regulation	0.040
Identified Regulation	0.420
Intrinsic regulation	0.616
Self-determination level	0.608

## Discussion

In terms of exploring the impact of “SPOC + flipped classroom” teaching on sports majors, the instructional intervention reduced *non-regulation*, *external regulation*, and *introjected regulation*, while increased *identified regulation*, *intrinsic regulation*, and *self-determination level* in the students. The levels of *non-regulation*, *external regulation*, *identified regulation*, and *self-determination* were also significantly different from those of the control group. This result is corroborated by many studies (Vallerand and Losier, 1999; Standage et al., 2006; Lonsdale et al., 2009; Alsancak Sirakaya and Ozdemir, 2018; Chuang et al., 2018), and some studies have suggested the promotion of “SPOC + flipped classroom” teaching in Russian higher education to improve motivational autonomy (Datsun, 2019). The aforementioned results show that “SPOC + flipped classroom” teaching promotes the transformation of motivation to internal motivation, that is, it promotes the internalization of motivation and improves the autonomy of motivation. This is related to the teaching design of the “SPOC + flipped classroom”. It provides an online SPOC platform for students to preview independently at any time before class, and in class, teachers answer the problems encountered by students’ self-study before class and carry out rich student-centered learning activities such as collaborative learning and group competition (Strayer, 2012). Students are in a learning environment that can meet their basic psychological needs, have a stronger sense of participation and identification with learning activities, and feel more attention and help from teachers and classmates. In addition, students are vulnerable to the influence of surrounding people when they engage in sports activities; the design of autonomous learning, group exploration, and teacher question answering of “SPOC + flipped classroom” teaching makes students pay less attention to the surrounding people and begin to pay attention to their own actual situation. Sports become an internal satisfaction and further promote the internalization of learning motivation. In addition, diversified teaching activities after class can further stimulate learning motivation and improve motivation autonomy (Botella et al., 2021; Faridah et al., 2021).

In terms of exploring the influencing factors and promotion paths of “SPOC + flipped classroom” teaching on sports students’ learning motivation, PLSR analysis results show that *demand for support* (*support for autonomy*, *support for relatedness*, and *support for competence*) and *basic psychological need satisfaction* (*need for competence* and *need for relatedness*) have a larger influence on learning motivation, and these variables can positively predict students’ *self-determination level*, *intrinsic regulation*, and *identified regulation*, and can negatively predict students’ *non-regulation*, *external regulation*, and *introjected regulation.* Emotion (*positive activity orientation*, *positive outcome orientation*, *negative activity orientation*, and *negative outcome orientation*) and the *need for autonomy* are the factors that have a great impact on learning motivation.

According to the *self-determination theory*, favorable external factors can have a positive effect on the internalization of motivation. When the external environment is more supportive of students’ autonomy, it will promote the development of students’ *intrinsic regulation* and *identified regulation*; otherwise, it will weaken the development of students’ autonomy regulation and may promote the development of *external regulation* and *introjected regulation* (Ryan, 1995; Ryan and Deci, 2000). Specifically, the “SPOC + flipped classroom” teaching method emphasizes student-centered teaching and combines online and offline teaching activities with information technology to provide a personalized learning environment for students, which is conducive to the internalization of students’ motivation to a certain extent. The model uses information technology to expand the time and space of teaching so that teaching has a greater space to play and provides richer learning activities, including after-the-class video learning, theoretical testing, video explanations, and other theoretical content; in-class exercises include competitions, displays, role plays, and other practical content so that students have more choices, which is conducive to reducing behavior control, developing autonomy adjustment, and improving motivation (Deci and Ryan, 1990; Vallerand and Losier, 1999). At the same time, the combination of online and offline learning links the inside and outside of class and gives timely feedback to each teaching activity. Sufficient before class preparation and positive feedback make students feel competent (Khayat et al., 2021). In addition, the model establishes a stable communication channel between teachers and students and provides a premise for enhancing the relationship between teachers and students. The process of this interactive cycle and the environment that includes online and offline integration inside and outside can make students perceive more needs for support (Long et al., 2017; Unal and Unal, 2017; Atkins, 2018) and further facilitates the adjustment and internalization of students’ learning motivation. Sanchez-Oliva et al. (2014) and Standage et al. (2006) found that the perception of teacher support predicts students’ autonomous motivation. The research of Sun and Ji (2010) also supports this conclusion and points out that teacher support can negatively predict *external regulation* and *non-regulation*, which strongly supports the practical results of this study.

*Basic psychological need satisfaction* is an important aspect of the *self-determination theory*, to achieve the high-quality development of motivation and the realization of optimal individual functions; three basic needs of individuals must be satisfied: *need for autonomy*, *need for competence*, and *need for relatedness* (Ryan and Deci, 2000; Garn et al., 2012). In the learning process, when the learning content is beyond the cognitive ability of students, students have a great cognitive load, and it is difficult to achieve a good learning effect, which may easily lead to frustration. This situation is changed in the teaching mode of “SPOC + flipped classroom” (Muir, 2021). Under the teaching mode of “SPOC + flipped classroom”, great changes have taken place in the teaching process and students’ cognitive process. Basic theoretical knowledge and the establishment of movement representation do not happen in class but are completed by students through video learning before class. While in class, concentrated physical practice is carried out based on the learning that took place before class. Learners’ previous knowledge reserves and various learning materials can reduce cognitive load (Paas and Van Merriënboer, 1994; Pollock et al., 2002), making students feel that the class content is “easier” and enhancing students’ sense of ability. In addition, before class learning will also provide a preview of the learning activities in class so that students are skilled in the exercise activities in class, and their sense of competence in learning activities is improved, which is beneficial to enhancing students’ autonomous motivation. Online platforms also provide a second place for communication between teachers and students, improving the frequency of interaction between teachers and students and making the teacher–student relationship closer so that students can be in a relaxed and pleasant psychological environment in the process of learning (Velde et al., 2021); these changes can better meet the psychological needs of students, thereby promoting the optimization of students’ learning motivation (Sergis et al., 2018; Zainuddin and Perera, 2019). The results of this study show that the *need for competence* and *need for relatedness* has a positive predictive effect on students’ autonomic motivation and negative prediction of students’ *non-regulation*, *external regulation*, and *introjected regulation*. Vasconcellos et al. (2020) conducted a meta-analysis of data from 265 related studies in the field of sports, and the results showed that *need for competence*, *need for autonomy*, and nee*d for relatedness* were significantly correlated with *intrinsic motivation*. Standage et al. (2005)’s research also highly supports this result. In addition, this study believes that there is a weak negative correlation between demand satisfaction and *introjected regulation*, which may be caused by different research scenarios and samples.

In summary, the teaching mode of “SPOC + flipped classroom” positively impacts the indicators of students’ motivation and promotes the level of autonomy of students’ motivation. “SPOC + flipped classroom” teaching enables students to obtain greater satisfaction by providing them more support, both of which promote the internalization of learning motivation so that students maintain a high level of autonomous motivation.

## Conclusion

Compared with traditional teaching, “SPOC + flipped classroom” teaching has a positive impact on students’ learning motivation of basketball skills and promotes students’ motivation autonomy. The improvement of *support for autonomy*, *support for competence*, *support for relatedness*, *need for competence*, and *need for relatedness* may be related to the mechanism of “SPOC + flipped classroom” teaching to improve the learning motivation of college students majoring in Physical Education (PE). “SPOC + flipped classroom” teaching enables students to obtain more demand satisfaction by giving them more demand support, while demand support and demand satisfaction can promote the internalization of learning motivation so that students can maintain high autonomy motivation.

## Limitations and prospects

From the perspective of the self-determination theory, this study analyzed and discussed the influence of “SPOC + flipped classroom” teaching on students’ motivation and its influence mechanism. After a semester of the teaching intervention, this research has made some achievements, but there are still some shortcomings, mainly reflected in the following aspects:

(1) During the research process, the COVID-19 epidemic interrupted the teaching intervention and disrupted the implementation of the research plan. The first teaching intervention was forced to be suspended, and the second teaching intervention was conducted after the pandemic ended; according to that, the research objectives and teaching objectives had to be adjusted, resulting in a smaller sample size.

(2) Due to the limitation of the number of research objects, the planned research methods (such as structural equation modeling) were not fully adopted in this study, and the analysis of the motivation influencing mechanism is still insufficient, which requires further research.

In future research, the aforementioned deficiencies should be addressed and improved. In addition, this study only analyzes technical courses in physical education. Future research can be carried out on theoretical courses of physical education and public physical education courses to analyze the similarities and differences and provide references and suggestions for improving the teaching of “SPOC + flipped classroom”.

## Data availability statement

The original contributions presented in this study are included in the article/supplementary material, further inquiries can be directed to the corresponding authors.

## Author contributions

TH: overall study design, and writing and revising manuscript. M-lZ: data collection and analysis, and manuscript writing. LC and HL: manuscript revision. All authors reviewed the manuscript, contributed to the article, and approved the submitted version.
